# Active Inference and Predictive Processing for Skill Learning: Implications for Skill Acquisition in Coaching Practice

**DOI:** 10.1002/ejsc.70292

**Published:** 2026-11-10

**Authors:** Jon Mackey, Dave Collins, Jamie Taylor

**Affiliations:** ^1^ Boxing Ireland National Stadium Dublin Ireland; ^2^ School of Health and Human Performance, Faculty of Science and Health Dublin City University Glasnevin Ireland; ^3^ Grey Matters Performance UK Stratford Upon Avon UK; ^4^ Moray House School of Education and Sport The University of Edinburgh Edinburgh UK; ^5^ CoEx|Lab, School of Health and Human Performance Dublin City University Glasnevin Ireland

**Keywords:** action‐oriented cognition, adaptive behaviour, motor learning, theory

## Abstract

This paper explores the potential of the Active Inference Framework (AIF) and Predictive Processing (PP) to advance our understanding of skill and motor learning with a key focus on introducing these concepts to coaches and highlighting their practical implications. Conceptual frameworks such as Information Processing (IP) and Ecological Dynamics (ED) have contributed significantly to the field of skill acquisition. Despite recent efforts to reconcile these perspectives, however, their fundamentally different theoretical foundations concerning perception, cognition and learning processes pose substantial challenges for integration. Although ED has made valuable contributions through its focus on perception‐action coupling and direct interaction with the environment, it involves adopting a radical ontological stance that emphasises rejecting traditional cognitive constructs that can complicate practical applications in coaching. Conversely, IP's reliance on concepts such as stored motor programs and the view of the brain as a computer‐like processor can limit its ability to account for the dynamic, emergent nature of skill acquisition in real‐world contexts. We suggest that AIF offers a parsimonious perspective, accommodating the dynamic, embodied and enactive nature of skill acquisition without the necessity of committing to either framework's specific assumptions. This perspective provides practitioners with a coherent and flexible framework for understanding and designing skill learning interventions, offering a practically useful account of skill acquisition for coaching practice.

## Highlights

- Active inference offers a way beyond framing skill acquisition as a polarised choice between information processing and ecological dynamics. Generative models provide an account of adaptive skilled behaviour that connects prediction with embodied perception and action.
- Scaling novelty and familiarity allows coaches to shape the challenge point through prediction errors. Mismatches between predicted and perceived sensory information can occur without execution mistakes, offering opportunities to explore movement solutions and develop skilled anticipation.
- Athlete understanding may shape expectations and support adaptive performance. Involving athletes in planning and helping them understand the purpose of practice may support learning.
- Coaches can use active inference to inform contextual judgement without adopting an ‘active inference coach’ identity. The framework's value lies in its explanatory utility.

## 1 Introduction

It has been suggested that the field of skill acquisition has been dominated by two polar‐opposite paradigms: Information Processing (IP) and Ecological Dynamics (ED). IP, emerging from mid‐20th‐century cognitive psychology, encompasses a range of theories that treat the brain as a processor of information, emphasising processes such as memory encoding, chunking and managing cognitive load to facilitate motor learning (Marteniuk 1976; Simon 1979; Sweller and Chandler 1991). In contrast, ED, rooted in Ecological Psychology and Dynamical Systems Theory, emphasises interaction with the environment through direct perception, emphasising attunement to affordances and the continuous coupling of perception and action as key mechanisms for skill acquisition (Araújo et al. 2006; Button et al. 2020).

Although IP and ED are often positioned as competing traditions, contemporary developments in both perspectives have softened some of the sharper oppositions. Our intention is therefore not to caricature either perspective but to highlight an explanatory challenge in the literature: how to account for adaptive skilled behaviour in sport. Practical recommendations emanating from IP and ED research share considerable overlap particularly in relation to variable practice, high‐fidelity or representative training design and the scaling of task complexity (Lindsay and Spittle 2024; R. D. Taylor et al. 2023).

The key distinction lies in how these approaches explain why such practices matter. Within IP, these strategies are typically understood as supporting the adaptation of sensorimotor transformations through the refinement of internal models that help predict and control action (Wolpert et al. 2001). In contrast, ED explains their value in terms of online coordination where behaviour emerges through nonlinear interactions among performer, task and environmental constraints (Renshaw et al. 2019). Thus, although practice design may look similar across the two traditions, the explanatory logic remains importantly different.

Instructional design approaches, guided by principles such as those from cognitive load theory, apply IP concepts by structuring tasks to effectively manage their cognitive demands (Rosenshine 2012; Sweller and Chandler 1991). In contrast, ED employs constraints‐led approaches (CLA) to create a representative design encouraging self‐organising behaviour (Araújo et al. 2007; Davids, Araújo, et al. 2003; Krause et al. 2019).

Importantly, theoretical differences do not always produce sharply contrasting coaching practice (Cassidy et al. 2025). In practice, if coaches were to adopt strongly theoretically driven practice, both IP‐ and ED‐informed stances would promote variability, representative tasks and reduced instruction. In these general cases, the difference is therefore less in practice and more in the underpinning explanatory logic.

Both IP and ED have faced theoretical and empirical critiques. Critiques of IP include suggestions that it is reductionist and conceptualises skilled behaviour as the execution of internalised templates or motor representations stored in the brain (Gottwald et al. 2023; Woods et al. 2020). Critiques in this regard have drawn on early Cognitivist notions portraying human behaviour as akin to a computer programme, where movements are pre‐planned and optimised through hierarchical processing stages. While frameworks like cognitive load theory provide valuable insights into structuring extrinsic information to reduce cognitive overload, they fall short in addressing how such information interacts with the continuous perception‐action loop central to skill learning (Seifert and Davids 2017). Ultimately, the key further limitation of IP lies in its treatment of perception and action as distinct, sequential processes. This dissociation contrasts sharply with ED's emphasis on the integrated and reciprocal nature of perception and action (Bruineberg et al. 2021). It also contrasts with embodied cognition perspectives, which position perception and action as mutually constitutive with movement requiring interaction with one's environment (Clark 1998). Furthermore, IP's staged models can oversimplify the complex and adaptive processes that underpin skilled behaviour in dynamic contexts.

Some critiques of IP accounts have argued that reliance on motor representations may be insufficient to fully explain skilled performance in context‐sensitive environments. For example, Hipólito et al. (2021) argue that skilful performance emerges not from executing internal instructions but from the flexible adaptation to environmental affordances. This critique only highlights the challenges posed when taking a top–down perspective on movement especially when applied to the context‐sensitivity of skilled behaviour. In contrast, ED frames such adaptability through concepts such as metastability, referring to a system's capacity to balance stability and flexibility (Kelso 2012), and affordances, understood as opportunities for action emerging in the performer‐environment relationship (Araújo et al. 2006). At the same time, it is important not to overstate this contrast, as later IP‐oriented work on prediction and internal modelling has also addressed issues of adaptive coordination and context sensitivity (e.g., Wolpert et al. 2001).

Additionally, the conceptual limitations of internal representations in IP have been challenged. Instructionist models, as critiqued by Hipólito et al. (2021), propose that skilful performance relies on explicit motor commands derived from detailed internal representations. In contrast, alternative frameworks that adopt a more interactionist approach suggest that skill emerges from embodied engagement with the environment rather than top–down motor planning (Bruineberg et al. 2021; Woods et al. 2020). These critiques suggest that IP, particularly in its classical formulations (e.g., Fodor 1983), faces significant challenges as a complete account of adaptive skilled movement. Whether these limitations remain in light of more recent predictive extensions of the IP tradition is contested.

Importantly, however, whilst the ED framework addresses many of the shortfalls of IP theory, it carries many of its own limitations. Many of these critiques have centred on Ecological Psychology's focus on organism‐environment interactions while overlooking sub‐personal, emergent processes and the failure to address the cognitive or neural underpinnings of perception (e.g., Read and Szokolszky 2020). Similarly, it has been suggested that affordances, as described in ED, are often treated as objective and pre‐existing features rather than relational and shaped by individual subjective experiences (Varela et al. 2017). This restricts the framework's ability to fully explain personalised, adaptive interactions with the environment (Read and Szokolszky 2020).

In practice, Kennedy and O’Brien (2024) suggest that the Constraints‐Led Approach, a framework for skill acquisition underpinned by ED that emphasises the interaction of task, environmental and individual constraints to guide the emergence of functional movement solutions through exploration and self‐organisation can be challenging to apply. This is due to the complexity of designing representative learning environments and the difficulty of standardising the measurement of emergent coordination. Indeed, empirical research on these approaches often struggles with issues of replicability and generalisability in real‐world settings, resulting in inconsistent and limited guidance for practitioners (Sheehan et al. 2022). Additionally, while CLA emphasises adaptability, inflexible application of the principles of encouraging self‐organisation and promoting exploration might lead to rigidity of coaching practices that overlook more cognitively focussed strategies (Bobrownicki et al. 2023).

Nonetheless, recent efforts to combine IP and ED perspectives have emerged from a practical need to accommodate diverse perspectives on skill acquisition within applied settings (Kearney et al. 2024; Lindsay and Spittle 2024). However, these attempts do not explicitly resolve the paradigms' underlying philosophical differences. The primary challenge remains in aligning IP's internal cognitive representations with ED's emphasis on nonrepresentational emergent behaviour shaped directly by environmental interactions (D. Harris et al. 2024). We would suggest that the issue is not merely whether one should side with IP or ED. Rather, it is whether existing explanations adequately capture the adaptive and context‐sensitive nature of skilled action.

## 2 Representation Wars—Framing the Problem

Fundamental differences between IP and ED theories lie in contrasting philosophical foundations, which reflect contrasting views of perception and cognition (D. Harris et al. 2024; Williams 2018). IP suggests that the brain constructs internal models of the external world. These models, or internal representations, are central to making sense of incoming sensory inputs, suggesting a sequential process wherein sensory inputs are processed before informing motor commands (Axelrod 1973; Marteniuk 1976; Simon 1979). In other words, perception and action are not tightly coupled; instead, action is considered an output following the processing of information.

In some literature and practice, this theoretical difference has sometimes led to the mistaken assumption that IP advocates for ‘low information’ skill learning, something that might be more closely associated with behaviourist approaches. For example, Optimal control theory, underpinned by IP, uses feedback loops to refine motor commands, where ongoing sensory feedback continuously updates movement execution, improving accuracy and updating long‐term memory (Stengel 1994; Todorov and Jordan 2002). Similarly, General Motor Programme Theory and Schema Theory rely on internal representations and memory structures, as they involve stored motor programs and schemas that guide movement production and evaluation based on past experiences and cognitive rules for adjusting actions (Schmidt 1975, 2014).

Jerry Fodor's modular theory of mind expanded the IP perspective (Fodor 1983). Fodor proposed that the brain is composed of specialised modules that process specific types of information independently, each constructing internal representations before passing them on for further cognitive processing. This view reinforced the idea of structured, domain‐specific cognitive mechanisms, suggesting that different aspects of perception and action are processed separately before being integrated into a cohesive response. Fodor's work relies on internal representations and sequential information processing, offering a more structured and compartmentalised view of cognition. Yet, theoretically at least, linear, sequential models of processing seem problematic.

Moving away from the representational stance, ED offers an alternative perspective, suggesting a direct, real‐time, continuous interaction with the environment, where perception involves detecting affordances—action possibilities directly available in the environment—without requiring internal cognitive processing (Bruineberg et al. 2018). This suggests that perception and action are tightly coupled with behaviour emerging from continuous interaction with the environment rather than cognitive processing (Button et al. 2020). Rejecting internal representations, ED asserts that perception is unmediated, relying on direct information available in the environment, which is said to provide all necessary information through the 'ambient optic array'—patterns of light interacting with objects that convey details without the need for mental reconstruction (Gibson 2002, 80). This stance is encapsulated by the claim that “the ecological commitment is to stand its ground: no representations, period” (Michaels and Palatinus 2014, 25).

Although ED is anchored in Ecological Psychology and Dynamical Systems Theory (DST), the two are not inherently linked. Gibson's work emphasised direct perception and affordances without initially relying on DST (Gibson 1961). DST, stemming from physics and mathematics, was later adopted to explain how movement patterns emerge from the interaction of various constraints, such as environmental, task‐related, and individual factors (Davids, Glazier, et al. 2003). Under the ED integration, DST connects the perception‐action coupling of Ecological Psychology with a proposal for the emergence of behaviour as self‐organising and grounded in attunement to affordances without reference to cognitive representations (Araújo and Davids 2004; Button et al. 2020; Davids, Glazier, et al. 2003).

These theoretical stances are polarised, not only within skill acquisition and motor learning (Ashford et al. 2021; Bobrownicki et al. 2023; Collins et al. 2022; Lindsay and Spittle 2024; R. D. Taylor et al. 2023), but more broadly across cognitive science (F. Adams 2018; Clark 2015a; Gärtner and Clowes 2017; Hipólito et al. 2021; Williams 2018). Given this deep‐rooted theoretical divergence, we argue that efforts to integrate these perspectives at a theoretical level seem a forlorn effort (for a similar position, see D. Harris et al. 2024). However, this is not to say that practitioners cannot adopt different lenses depending on their coaching context (Collins and Taylor 2025).

## 3 Predictive Processing, an Embodied Framework for Perception and Action

Promising recent developments in the fields of neuroscience, neurobiology and cognitive science present a set of theories under the umbrella of Predictive Processing (PP). These include the Bayesian Brain Hypothesis (Colombo and Seriès 2012; Knill and Pouget 2004), Predictive Coding (Hohwy 2013) and, pertinent to this paper, the Active Inference Framework (AIF) (Friston et al. 2017). Each of these aims to offer a path towards resolving the so‐called 'representation wars’ (Constant et al. 2021). As a consequence, and based on our practical intention, we first introduce PP and the AIF to the interested coach and, by drawing on the theoretical literature, tentatively propose implications for coaching practice.

PP is an embodied and enactive cognitive framework that explains how an organism processes sensory information by continually predicting and adjusting expectations about incoming stimuli (Bruineberg et al. 2018; Clark 2013, 2015b; Sprevak and Smith 2023). Embodied refers to the view that cognition is shaped by the body's neural and extra‐neural processes. Enactive refers to the view that cognition arises through perceptually guided engagement with the world (Gallagher 2023).

PP is rooted in the work of 19th‐century scientist Hermann von Helmholtz, who proposed that perception is essentially an act of unconscious inference (Barlow 1990; Patton 2022). According to this view, the brain generates predictions about the external world based on past experiences, shaping internal models that guide perception and action in light of the anticipated sensory consequences. By reducing the difference between predicted and perceived states, the system can perpetuate itself and guide adaptive action.

These predictions are continuously tested against various sensory data, such as visual, auditory, proprioceptive, interoceptive and haptic information. The discrepancies between predictions and perceived sensory data, known as prediction errors, prompt adjustments, not just in neural processes but across the brain/body system, refining the organism's engagement with its environment. Contrasting with the day‐to‐day use of the term, rather than a mistake, prediction *error* refers to the difference between the organism's predictions and perceived sensory input. The system's goal is to minimise prediction error by either updating dynamic models (perceptual inference) or acting to align the world with these predictions.

The concept of precision weighting plays a crucial role in this process (Friston et al. 2017), governing how prediction errors are processed by modulating the influence of sensory inputs and prior beliefs. Precision reflects the estimated reliability of information with high sensory precision emphasising bottom–up data and high prior precision favouring top–down predictions. In a practical example, using the case of catching a fly ball in baseball, the athlete's continuous interaction with the environment enables the real‐time adjustment of their body based on the concept of active sensing where perception and action are tightly coupled in a dynamic feedback loop. Rather than passively observing and computing a detailed internal model, the outfielder engages in embodied problem‐solving by moving so as to cancel the ball's optical acceleration (i.e., keeping its apparent rise steady). Read through AIF, this strategy enables the outfielder to predict using precision weighting and adjust their actions in real time, ensuring they reach the landing spot (Clark 2024b; Hipólito et al. 2021).

## 4 Active Inference

The AIF is grounded in the principles of 4E cognition—embodied, embedded, enactive and extended (Menary 2010). This may offer a way to reconcile the key tensions of cognitive science in practical applications. To understand this potential, it is necessary to unpack the relationship between 4E cognition and the AIF. Embodied cognition suggests that cognitive processes are rooted in the physical body. Embedded cognition situates these processes within a broader environmental and social context (Varela et al. 2017). Enactive cognition highlights the dynamic action‐oriented interactions between an organism and its environment (Hutto and Myin 2017), and extended cognition suggests that cognitive processes can extend beyond the organism to include tools and other external structures (Clark and Chalmers 2010). For example, the bike may become an extension of the cyclist. Together, these principles challenge internalist and computational views of cognition by focussing on the agent‐environment system.

AIF integrates 4E principles by accommodating internal modelling processes and continuous interaction with the environment. Specifically, it proposes that cognition involves a dynamic balance between minimising prediction errors via a generative model and actively sampling the environment to confirm or adjust those predictions (Constant et al. 2021; Friston 2010). By embedding models within the broader context of embodied and enactive interactions, AIF captures the situated, adaptive nature of cognition while retaining the explanatory power of modelling. For example, the generative models of AIF are not static representations but dynamic, action‐oriented structures that continuously evolve through organism‐environment interactions (Constant et al. 2021; Kiverstein et al. 2019).

This dual focus on internal modelling and ongoing engagement with the environment positions AIF as a framework that may help bridge the apparent divide between representational and nonrepresentational perspectives (Constant et al. 2021). At the heart of AIF is the idea that all living things aim to reduce surprise or uncertainty about what they sense in their environment (Bruineberg et al. 2018; Friston 2010). The Free Energy Principle suggests that living organisms aim to maintain stability by reducing uncertainty, acting to minimise discrepancy between expected and perceived sensory input and continuously updating to reduce 'surprise' or unpredictability (Friston et al. 2016). While Helmholtz emphasised perception as an inferential process, AIF goes further by incorporating action into the predictive model. Here, the organism does not merely infer the state of the world but actively interacts with it to confirm its hypotheses and reduce uncertainty (Shestakov et al. 2022). Organisms minimise uncertainty by constantly updating generative models based on interactions with their environment (Feldman and Friston 2010; Friston 2010; Friston et al. 2017). Rather than treating perception and action as separate processes, AIF integrates them as *interdependent* processes, dynamically adjusting behaviour based on sensory feedback to enhance adaptation and learning (Friston et al. 2016).

In this view, learning involves the ongoing refinement of generative models, which are not static but dynamic patterns that emerge through embodied engagement with the world (Bruineberg et al. 2018; Friston et al. 2017). Generative models create top–down predictions about sensory input, allowing the organism to interpret and respond to its environment more efficiently. By constantly adjusting these predictions, based on the balance between prior expectations and new evidence, the predictive system learns and adapts (D. J. Harris et al. 2022; D. J. Harris et al. 2023). Generative models are considered to be hierarchical, where higher‐level predictions inform lower‐level sensory processing and vice versa. Hierarchical models are layered with higher and more abstract predictions influencing lower‐level predictions.

When there is a discrepancy between prediction and what is perceived at lower levels, the prediction error is generated and sent upward in the hierarchy. Hierarchical generative models create a predictive structure where each level refines the levels above and below it. This process allows for efficient and adaptive use of sensory data, with predictions shaped by both the immediate context and broader, abstract expectations. For example, R. A. Adams et al. (2013) propose a shift in understanding motor control, arguing that it does not rely on descending motor commands but on proprioceptive predictions that minimise prediction errors. This approach reinterprets classical motor reflexes and the functional anatomy of the motor system as mechanisms for fulfilling proprioceptive predictions rather than executing pre‐planned commands. Thus, AIF serves as a promising framework for advancing our understanding of cognition, providing a nuanced account that is sensitive to both internal mechanisms and the lived, relational dynamics of the organism in its environment.

### 4.1 Ecological Perception

Linson et al. (2018) present AIF as extending beyond the earlier critiqued linear sequential processing model, instead involving continuous integration between an individual and their environment. This positions perception and action as parts of a dynamic loop, integrating exteroception (external information), proprioception (body position and movement) and interoception (the body's internal state), allowing for the adaptation of behaviour. This mirrors elements of ED in its recognition of perception‐action coupling (Bertenthal et al. 1997) and broadens the Gibsonian concept of affordances by extending their scope to include a broader spectrum of sense data (Linson et al. 2018). Rather than directly perceiving affordances as invariant properties of the environment, AIF treats affordances as anticipatory and generative. That is, affordance refers to the opportunities for action that arise based on an organism's generative model of its environment and its goals (Friston 2022).

This predictive engagement goes beyond Gibson's real‐time affordance‐response interaction by incorporating prior information with predictions and aligning perception and action more closely with the generative model. Thus, AIF views affordances as opportunities for action selected to minimise free energy, balancing epistemic (information‐seeking) and pragmatic (goal‐oriented) values.

In practice, this means that athletes continuously predict the most relevant affordances, such as a forward in soccer anticipating the pass of a defender before initiating a press, something referred to in soccer as a ‘pressing trigger’. Similarly, a boxer may anticipate an opponent's punch based on prior movements or kinematic cues and adjust their position or counterattack to minimise prediction error. For example, a fighter watching clips of an opponent might study their counter‐punching techniques to gather information (epistemic value) about tendencies and timing. Here, the epistemic aspect involves gathering information to improve predictions, while the pragmatic aspect refers to using this knowledge to guide real‐time actions in the ring. AIF therefore suggests that athletes actively infer possible outcomes and select actions that maximise the accuracy of their generative models, reducing uncertainty and improving performance (R. A. Adams et al. 2013; Cappuccio et al. 2020).

## 5 Three Core Implications for Coaching Practice

Another important feature of AIF is the aim to both accommodate existing theories and embrace theories that work under a unified framework (Parr et al. 2022). As such, if AIF does provide our best current explanation for human functioning, there is no need to ‘throw the baby out with the bath water’. This allows us to consider what existing features of coaching seem to be most coherent with the AIF.

### 5.1 Developing Athlete Understanding

AIF presents a structured approach to understanding action and motor learning, grounded in the idea that behaviour is organised through the organism's efforts to minimise prediction error. One of the clearest practical implications from AIF is the potential for us to leverage expectancy effects in coaching. Placebo effects, for example, demonstrate how expectations can actively shape physiological and psychological outcomes even when the intervention itself lacks direct causal efficacy (Clark 2024a). By fostering confidence and creating positive expectations, coaches can tap into these mechanisms to enhance performance and well‐being, aligning with the brain's natural propensity to minimise prediction error. In this sense, it would seem important that coaches both involve athletes in the planning of their training (recently referred to as co‐construction) and, perhaps more importantly, help them to articulate *why* they are doing what they are doing. In essence, it would suggest that adaptation to training stimulus may be enhanced as a result of understanding, which coaches perceive as being critical for adaptation (Anyadike‐Danes et al. 2023) while also enhancing performance under pressure (McKay et al. 2012).

Another form of understanding relates to awareness of movement. The AIF, and specifically the generative hierarchical model, may provide the theoretical ground for understanding what has historically been referred to as the conscious control of movement (Masters 1992). The notions of automaticity and implicit learning are controversial areas in skill acquisition (McKay and Carter 2023). In addition to the understanding of why someone might be training in a particular way, we speculate on how understanding of movement at more abstract levels of a generative hierarchy may enhance athlete adaptability in action. When an athlete practises a skill, AIF suggests that the brain/body system is not just responding to the sensory input but actively predicting the outcomes of movements, updating its expectations to better align with actual performance (Friston et al. 2011; Proietti et al. 2023). This involves hierarchical inference, spanning different levels of action understanding—from basic kinematics to broader goals and intentions (e.g., tactics). As another example, in tennis, recognising the movement patterns associated with a forehand shot enables the athlete to infer its purpose, such as driving the ball cross‐court to exploit an opponent's weak side (Proietti et al. 2023).

For the time being, the AIF may offer grounds for explaining existing theory. One example is the Multi‐Action Plan (MAP) model, which has been used to explain how athletes regulate perception, action and transitions between mental states during performance (Kellermann et al. 2022). MAP categorises performance into four distinct types: T1 (automatic optimal performance), T2 (controlled yet effective performance), T3 (effortful and suboptimal performance) and T4 (automatic yet suboptimal performance) (Kellermann et al. 2022, 2024). Drawing on the AIF, this would suggest that when prediction error is low, the athlete may be in an optimal performance state (T1). However, as prediction error increases, the athlete may experience more controlled or effortful states (T2 or T3) as errors are pushing further up the generative hierarchy to more abstract levels.

Under high pressure, the experience of high uncertainty and prediction error may shift the athlete from a smooth, relatively automatic performance (T1) into a more effortful state in which additional resources are allocated to update the generative model. This transition may take at least two forms, both of which involve conscious control of action. In T2, attention is directed towards individually functional components of the action that are causally linked to success. In T3, attention becomes dysfunctional with attention directed towards the conscious regulation of otherwise integrated movement (Carson and Collins 2016).

For example, for a canoe slalom paddler navigating a difficult section of a course in an optimal state (T1), the generative model accurately anticipates the actions required, enabling fluent execution. Under pressure, however, rising prediction error may prompt a transition to a more effortful state. In a T2‐like state, this may involve consciously attending to the timing of the catch relative to an upstream gate. In a T3‐like state, the paddler may attempt to consciously regulate arm position, trunk rotation and blade angle separately, potentially disrupting coordination.

### 5.2 Skilled Anticipation and Prediction

In the context of sport, anticipation is one of the hallmarks of expertise (Farrow and Abernethy 2015). Under the AIF, anticipation is underpinned by active engagement with the environment, where the brain/body system uses predictive models to prepare for various possibilities. Skilled performers are adept at leveraging these models to maintain performance even in uncertain conditions. Under the AIF, we can account for the interaction between an agent and their environment, using existing definitions of skilled movement, such as that of Bennett and Fransen (2024) “the adaptation of one's technique to produce functional and beneficial outcomes in contexts that require them” (p. 398). In this sense, AIF allows for a consideration of the capacities of the agent, how sense data is used for predictive control of movement and ultimately, the extent of their grip of the environment (Kiverstein et al. 2019).

Skilled athletes dynamically identify and act on affordances using anticipatory mechanisms to guide their interaction with the environment (Abernethy et al. 2012; Farrow and Abernethy 2015; Müller and Abernethy 2012). According to Cappuccio et al. (2020), such anticipatory mechanisms involve both explicit cues from the environment and internal simulations based on prior experience, which allow for quicker and more accurate responses (see also Abreu et al. 2017). This highlights how skilled athletes can generate accurate predictions in dynamic environments (D. J. Harris et al. 2022). This is especially evident in fast‐paced sports such as tennis or boxing where athletes must predict their opponents' actions and adapt their actions accordingly. This is also the case in team sport, where athletes need to predict the actions not only of opponents but also of teammates. Indeed, in both circumstances, highly skilled performers will not only anticipate but act proactively to ‘encourage’ actions from opponents that fit their a priori developed plans.

The consequence for coaches is the need to ‘coach for prediction’, which involves creating dynamic learning environments that encourage athletes to explore, expand and refine their ability to anticipate and respond to, or even to shape, their opponents' challenges. By designing tasks that balance novelty and familiarity, coaches can shape athletes' experiences according to intention (J. Taylor and Ashford 2025). This enables coaches to moderate the challenge point (cf. Guadagnoli and Lee 2004) to a level where progress in reducing prediction error is both achievable and rewarding (Kiverstein et al. 2019). The coaching style adopted by the coach therefore plays a critical role in moderating the extent of the prediction error experienced and keeping it within a desired bandwidth. Moreover, athletes can be supported to recognise emotional responses as useful information to shape movement solutions. Consequently, coaches need to interpret these responses in psycho‐motor and psycho‐social terms (Ferguson et al. 2025).

Changes in ball speed, trajectory or surface can create prediction errors that the athlete needs to resolve, promoting motor learning and improving their predictive capacity for competition (Friston et al. 2016). This may also involve applied methods long advocated in the literature, such as the explicit coaching of task‐relevant cues (e.g., Vickers 2007) and the use of imagery to prime and rehearse for competition (Collins and Carson 2017).

### 5.3 Further Acknowledging the Centrality of Error to Learning and Performance

Whilst acknowledging that prediction error is different from the lay use of the term, what AIF should draw our attention to is the centrality of the difference between prediction and perceived sense data as the source of learning. Errors, understood as mismatches between expectations and outcomes, drive adaptation by signalling adjustments in action or beliefs. As has been acknowledged for decades (e.g., Guadagnoli and Lee 2004), rather than something to avoid, errors are important for refining and stabilising skilled action.

Additionally, error‐based learning drills are beneficial where the training conditions intentionally introduce discrepancies between expected and actual outcomes, such as contrast drills to perturb stable movement patterns, or using progressive imagery techniques to challenge athletes' habitual motor routines and encourage exploratory adjustments (Carson and Collins 2015).

By engaging in tasks that promote the resolution of uncertainty and the refinement of action, athletes can enhance their adaptive responses and generalise skills across varying scenarios (Friston et al. 2016; O’Brien et al. 2024). For example, practicing the same tennis shot under various lighting conditions can help the athlete learn to cope with changes in visual information, strengthening their ability to perform consistently in competition.

## 6 Conclusion

In this paper, we have introduced the Active Inference Framework as a theoretical underpinning that may help the motor learning and skill acquisition literature to move beyond the dichotomised perspectives between Information Processing (IP) and Ecological Dynamics (ED) theories. AIF provides a unified framework that integrates cognitive processes as embodied and enactive interactions with the environment, as a continuous dynamic loop between perception and action. Whilst there remains little empirical work in sport using AIF as a theoretical lens, we have suggested key implications for the sport coach. These included leveraging the AIF concept to design learning environments that emphasise adaptive, exploratory behaviours. Coaches are encouraged to create conditions that promote the resolution of prediction errors, fostering athletes' ability to refine and enhance skill generalisation. AIF also highlights the importance of skilled anticipation, suggesting that training should involve dynamic, representative tasks that help athletes anticipate and respond to challenges by actively engaging with their environment. Additionally, the framework underscores the centrality of error‐based learning, where discrepancies between predictions and outcomes drive skill refinement. Practical strategies, such as co‐construction of training plans, adaptive coaching styles, and incorporating action observation and simulation, are recommended to enhance practice based on the theoretical principles advocated by the AIF.

Furthermore, in the literature, there is increasing discussion regarding the need for coaches to adopt a single perspective to inform their work. Whilst we see genuine theoretical promise for the AIF to influence the progression of sport coaching, we explicitly do not suggest that coaches begin to adopt the label of being an ‘active inference coach’. Instead, in acknowledgement of the social complexity of coaching practice (Cushion 2007), we take a pragmatic, fallibilistic view (Collins et al. 2022), one that acknowledges both the theoretical promise of AIF and the absence, thus far, of empirical application in sport coaching contexts.

## Funding

The authors have nothing to report.

## Conflicts of Interest

The authors declare no conflicts of interest.

## Data Availability

The authors have nothing to report.
